# Integrated Metabolomic and Transcriptomic Analysis and Identification of Dammarenediol-II Synthase Involved in Saponin Biosynthesis in *Gynostemma longipes*

**DOI:** 10.3389/fpls.2022.852377

**Published:** 2022-03-25

**Authors:** Shuang Ye, Lei Feng, Shiyu Zhang, Yingchun Lu, Guisheng Xiang, Bo Nian, Qian Wang, Shuangyan Zhang, Wanling Song, Ling Yang, Xiangyu Liu, Baowen Feng, Guanghui Zhang, Bing Hao, Shengchao Yang

**Affiliations:** ^1^State Key Laboratory of Conservation and Utilization of Bio-Resources in Yunnan, The Key Laboratory of Medicinal Plant Biology of Yunnan Province, National & Local Joint Engineering Research Center on Germplasms Innovation & Utilization of Chinese Medicinal Materials in Southwest China, Yunnan Agricultural University, Kunming, China; ^2^Centre for Mountain Futures, Kunming Institute of Botany, Kunming, China; ^3^Center of Excellence in Fungal Research, Mae Fah Luang University, Chiang Rai, Thailand; ^4^Honwin Pharma (Lianghe) Co., LTD., Dehong, China

**Keywords:** gypenosides, oxidosqualene cyclase, cytochrome P450 monooxygenase, dammarenediol II synthase, *Gynostemma longipes*

## Abstract

*Gynostemma longipes* contains an abundance of dammarane-type ginsenosides and gypenosides that exhibit extensive pharmacological activities. Increasing attention has been paid to the elucidation of cytochrome P450 monooxygenases (*CYP*s) and UDP-dependent glycosyltransferases (*UGT*s) that participate downstream of ginsenoside biosynthesis in the *Panax* genus. However, information on oxidosqualene cyclases (*OSCs*), the upstream genes responsible for the biosynthesis of different skeletons of ginsenoside and gypenosides, is rarely reported. Here, an integrative study of the metabolome and the transcriptome in the leaf, stolon, and rattan was conducted and the function of *GlOSC1* was demonstrated. In total, 46 triterpenes were detected and found to be highly abundant in the stolon, whereas gene expression analysis indicated that the upstream *OSC* genes responsible for saponin skeleton biosynthesis were highly expressed in the leaf. These findings indicated that the saponin skeletons were mainly biosynthesized in the leaf by *OSC*s, and subsequently transferred to the stolon via *CYP*s and *UGT*s biosynthesis to form various ginsenoside and gypenosides. Additionally, a new dammarane-II synthase (*DDS*), *GlOSC1*, was identified by bioinformatics analysis, yeast expression assay, and enzyme assays. The results of the liquid chromatography–mass spectrometry (LC–MS) analysis proved that *GlOSC1* could catalyze 2,3-oxidosqualene to form dammarenediol-II via cyclization. This work uncovered the biosynthetic mechanism of dammarenediol-II, an important starting substrate for ginsenoside and gypenosides biosynthesis, and may achieve the increased yield of valuable ginsenosides and gypenosides produced under excess substrate in a yeast cell factory through synthetic biology strategy.

## Introduction

Ginsenosides are valuable natural compounds found throughout species of the *Panax* genus that have the ability to treat cardiovascular and cerebrovascular diseases (Liu et al., 2017). Based on the different skeletons, three types of ginsenoside have been found in the *Panax* genus: dammarane-, ocotillo-, and oleanane-type ginsenosides (Christensen, 2008; Hou et al., 2021). Previous studies have shown that dammarane ginsenosides exist only in the *Panax* genus, such as *Panax ginseng* (ginseng), *Panax quinquefolium* (American ginseng), and *Panax notoginseng* (Sanqi), which are the three main species of the *Panax* genus cultivated worldwide. The global market demands for this high-value medical plant cannot be met due to the 4- to 7-year planting cycle and low yield (He et al., 2019).

Species in the *Gynostemma* genus, a member of the Cucurbitaceae family, have similar dammarane ginsenoside content to those in the *Panax* genus (Gantait et al., 2020). *Gynostemma longipes* has the highest content of dammarane ginsenoside, reaching 25%, which is even higher than *Panax* plants (Razmovski-Naumovski et al., 2005). Gypenosides in *G. longipes* have panaxadiol and 2α-hydroxypanaxadiol skeletons, which are similar to those of dammarane ginsenosides and have similar pharmacological effects, such as anticancer, cardioprotective, hepatoprotective, neuroprotective, and anti-inflammatory activities (Nagai et al., 1976; Li et al., 2019; Nguyen et al., 2021; Su et al., 2021). Unlike the medicinal root of *Panax* genus, the main medical parts of *G. longipes* are the stolon and the leaf, which can be harvested for many years from a single planting, indicating it was more convenient to obtain dammarane-type saponins from *Gynostemma* plants than from *Panax* species. *G. longipes* has become an alternative plant to support the deficiencies in the ginsenoside extract market (Ky et al., 2010). In China, 3.2 billion tons of *G. longipes* are processed for pharmaceutical extraction, with an annual production of 803 billion RMB in 2018 (Cheng and Cheng, 2019).

**FIGURE 1:**
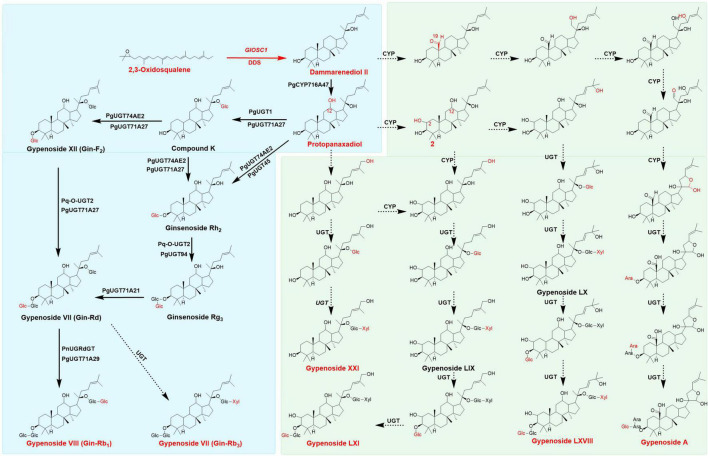
Proposed Biosynthesis pathway of gypenosides and ginsenosides, the main triterpenes were colored in red, the broad arrows presented the enzymes already identified, dotted arrows presented the assumed enzymes in gypenosides analysis. Pathways in green background only found in *Gynostemma* plants, pathways in blue back ground could appear both in *Gynostemma* plants and *Panax* plants.

In the past decade, researchers have employed synthetic biology techniques to produce valuable natural compounds from microorganism that are difficult to product through chemical synthesis to satisfy the demands of the market. The ginsenosides R0, Rh2, Rg3, Rg1, and Rb1 have been biosynthesized in *Escherichia coli* and yeast (Wang et al., 2015; Lu et al., 2018; Tang et al., 2019, 2021). Previous studies have described the biosynthesis of dammarenediol-II from 2,3-oxidosqualene under the catalysis of dammarenediol-II synthase. *PgCYP716A47* catalyzed the hydroxylation at the C-12 of dammarenediol to form protopanaxadiol (Han et al., 2011). After the formation of the protopanaxadiol skeleton, UDP-dependent glycosyltransferases add glucose to different positions to generate various ginsenosides: *Pq-O-UGT1*, *PgUGT45*, or *PgUGT74AE2* catalyzed the glycosylation of protopanaxadiol to form ginsenoside Rh2; *Pq-O-UGT2* and *PgUGT94* catalyzed Rh2 to form Rg3 (Yang et al., 2020); Rd was formed under the catalysis of *PgUGT71*; *PgUGT1* and *PgUGT71A27* catalyzed protopanaxadiol to form compound K (CK), which was subsequently catalyzed by *PgUGT74AE2*, *PgUGT71A27*, *Pq-O-UGT2*, and *PgUGT71A27* to produce F2 and Rd. *PgUGTdGT* and *PgUGT71A29* could utilize Rd as substrate to generate Rb1 (Yang et al., 2018; Figure 1). These ginsenosides mentioned above were found in *Gynostemma* plants, indicating *Gynostemma* was the best resource for ginsenosides after *Panax* plants (Qin et al., 1992).

The genes involved in biosynthesis of gypenosides were remain large unknown, few studies analyzed and predicted the possible candidate genes involved in gypenosides biosynthesis in *G. pentaphyllum* (Huang et al., 2021; Zhang et al., 2021). Recently, 5 UDP-glycosyltransferases were elucidated in ginsenoside biosynthesis which could form F1, Rh2, Rg3, CK, F2 and Rd with UDP-glucose as sugar donor in *G. pentaphyllum* (Le et al., 2021). The biosynthesis of gypenoside starts with the formation of 2,3-oxidosqualene through the mevalonate (MVA) pathway in the cytoplasm and the methylerythritol phosphate (MEP) pathway in plastids. 2,3-Oxidosqualene forms different triterpenoid skeletons under the catalysis of oxidosqualene cyclase (*OSC*s), which are subsequently modified by *CYP*s and UDP-dependent glycosyltransferases (*UGT*s), forming different ginsenosides (Kim et al., 2015; Yang et al., 2018). However, compared with ginsenosides, the structural diversity of gypenosides was more abundant owing to multiple levels of oxidation (CH_2_OH or CHO) at C-2 and C-19 sites, as well as the side chains in gypenosides (Figure 1; Yoshikawa, 1986; Razmovski-Naumovski et al., 2005; Nguyen et al., 2021). However, the specific genes involved gypenoside biosynthesis and their functions remain largely uncharacterized.

To elucidate the biosynthesis pathway of gypenosides in *Gynostemma* plants, an integrated analysis of transcriptomics and metabolomics was performed in this study to provide more information on various specific genes and gypenosides. The solon and leaf of *Gynostemma longipes*, the main resource for commercial gypenoside extraction in China, were collected and analyzed by high-throughput omics techniques. Three non-steroidal-type triterpene synthases that catalyze oxidosqualene through dammarenyl cations were found after comparison of amino acids and phylogenetic analysis. Twelve candidate *CYP*s were found to participate in gypenoside biosynthesis by performing gene co-expression analysis between *CYP*s and triterpenes. Moreover, a dammarenediol-II synthase that catalyzed 2,3-oxidosqualene to form dammarenediol-II was found and verified in yeast. These results provide insight into gypenoside biosynthesis in *Gynostemma* plants to allow better development of gypenosides using synthetic biology techniques.

## Results

### Illumina Sequencing and *de novo* Assembly

cDNA libraries were constructed from the total RNA of the leaf, rattan, and stolon to characterize transcriptome of *G. longipes*. Three biological replicates of each tissue were processed and sequenced on an Illumina Hiseq™ 2000 platform. In total, 438,908,642 (65,385,856,136 bp) clean reads were generated with 44.5429% GC after filtering out adaptor sequences, ambiguous reads, and low-quality reads (Q20 < 20). All clean reads were assembled into 1,290,066 unigenes within a total length of 96,791,710 bp. The number order of unigenes contig N50 was 23114; the length was 1184 bp. The size of unigenes ranged from 201 bp to 16470 bp with an average length of 749 bp (Table 1). The high-quality reads in this research have been deposited in NCBI SRA database (accession number: PRJNA784129).

**TABLE 1:** Summary of Illumina sequencing and assembly of *G. longipes.*

Database	Number of unigenes	Annotation percentage
Nr	78398	60.74%
KEGG	66100	51.21%
COG	49036	37.99%
Swiss-Prot	58300	45.17%
Annotated in four databases	41791	32.38%
All annotated	81721	63.32%
Total unigenes	129066	

### Gene Annotation and Expression Analysis

BLASTx was applied to annotate unigenes based on four databases: NCBI non-redundant protein (Nr), Kyoto Encyclopedia of Genes and Genomes (KEGG), the Clusters of Orthologous Groups/Clusters of orthologous groups for eukaryotic complete (COG/KOG), and the Swiss-Prot protein database. In total, 81,721 (63.32%) unigenes were annotated; among them, 78,398 (60.74%) unigenes were matched in the Nr database, 66,100 (51.21%) unigenes were matched in the KEGG database, 49,036 (37.99%) unigenes were matched in COG/KOG database, and 58,300 (45.17%) unigenes were matched in the Swiss-Prot database. Overall, 41,791 (32.38%) unigenes were matched in all databases (Table 2).

**TABLE 2:** Summary of annotation of *G. longipes* unigenes.

Database	Number	Length (bp)
Total raw reads	439725724	65958858600
Total clean reads	438908642	65385856136
GC percentage (%)	44.529	
Contigs N50 number	23114	1184
Number of unigenes	129066	96791710
Average length of unigenes (bp)	749	
Max length of unigenes (bp)	16470	
Min length of unigenes (bp)	201	

The comparison of unigenes from the three tissues identified 4883, 2374, and 4480 unigenes specifically annotated in the rattan, stolon, and leaf, respectively, with 15,015 unigenes annotated in all tissues (Supplementary Figure S1B). The results showed that 59,894 unigenes were classified in biological process ontology (GO: 008150), the major terms were metabolite process (15,454, 25.80%), cellular process (12,824, 21.41%), and single-organism process (9791, 16.35%); 26,224 unigenes belonged to cell component ontology (GO:0005575) (Supplementary Figure S1A). In this ontology analysis, catalytic activity (13,234, 50.47%) and binding (10380, 39.58%) were the major terms; 43,190 unigenes belonged to molecular function ontology (GO: 003647), cell (10701, 24.78%), cell part (10687, 24.74%), and organelle (6710, 15.53%) (Supplementary Figure S1C). Moreover, based on KEGG annotation, 8877 unigenes were divided into five categories, including organism system, cellular processing, environment information process, genetic information process, and metabolism. It is notable that 491 unigenes (3.5% of the metabolism category) participated in the metabolism of terpenoids and polyketides (Supplementary Figure S1A). These data provided basic information for mining candidate genes involved in gypenoside biosynthesis. The original data of RNA-seq annotation and expression could be downloaded in Medicinal Plants multi-Omics Database^1^ (He et al., 2022).

### Analysis of Triterpene Content and Gene Expression Profile in *Gynostemma longipes*

**FIGURE 2:**
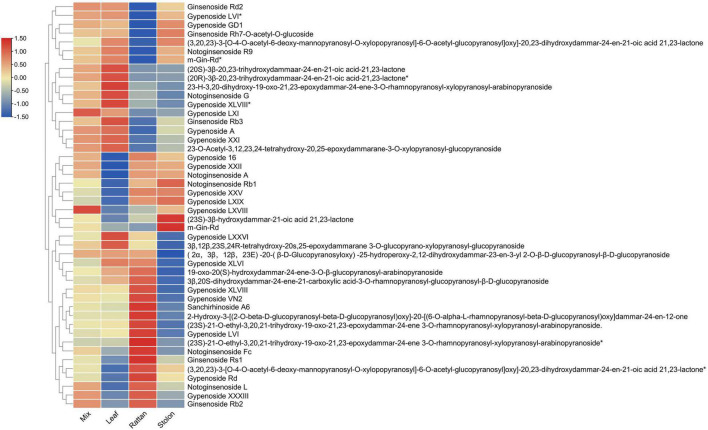
The heatmap of triterpene content level in leaf, rattan, stolon and whole plant of *G. longipes*.

The metabolomic analyses of the leaf, stolon, and rattan detected 46 triterpenes in *G. longipes*. Seventeen triterpenes (ginsenoside Rd2 to 2-3-*O*-acetyl-3,12,23,24-tetrahydroxy-20,25-epoxydammarane-3-*O*-xylopranosyl-glucopyranoside) had the highest content in the leaf, followed by the rattan (*indicates isomers exist). For nine triterpenes (from gypenoside 16 to m-Gin-Rd), the content was higher in the rattan than in the stolon. The relative content of 20 triterpenes in the stolon was higher than in the rattan and the leaf (from gypenosides LXXVI to ginsenoside Rb2). The results showed that the content of 29 triterpenes was relatively high in the stolon, suggesting it might be a potential tissue in which triterpene accumulation occurs (Figure 2). Otherwise, the relative contents of gypenoside LXI, gypenoside A, and gypenoside LXVIII were relatively high in the whole plant, indicating these two triterpenes may be the main triterpenes in *G. longipes*.

**FIGURE 3:**
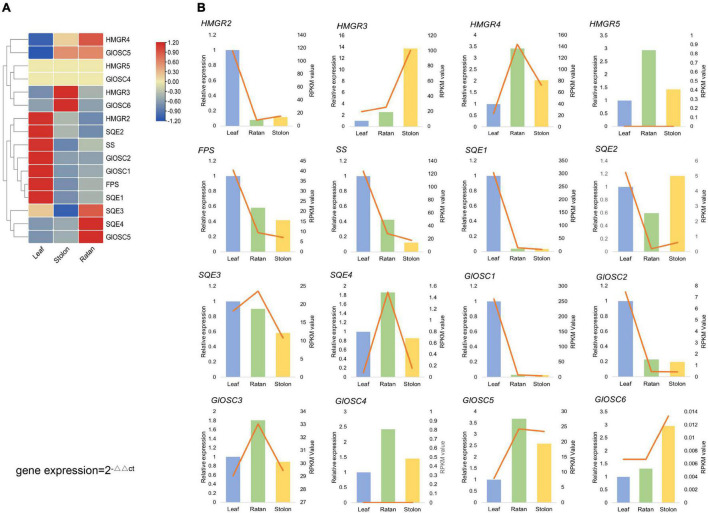
**(A)** Expression analysis of the candidate genes, expression level of *HMGR*s, *GlOSC*s, *SS*s, *FPS*, *SE* in leaf, rattan and stolon of *G. longipes.* **(B)** Real-time quantitative polymerase chain reaction (PCR) validation of candidate unigenes involved in gypenosides biosynthesis by RNA-seq. The histogram indicated the relative gene gypenosides biosynthesis by RNA-seq, the right *y*-axis was gene expression level calculated as RPKM value, the left *y*-axis was relative gene expression obtained via real-time PCR. The relative expression obtained from real-time PCR calculated by 2^–ΔΔCt^ method.

From the transcriptome analysis, 16 genes were considered to encode key enzymes involved in triterpene biosynthesis through the MVA pathway: four *HMGR*s (3-hydroxy-3-methylglutaryl-coenzyme A reductases), one *FPS* (farnesyl diphosphate synthases), one *SS* (squalene synthase), four *SQE*s (squalene epoxidases), and six *OSC*s (2,3-oxidosqualene cyclases). The expression of *SS*, *FPS*, *HMGR2*, *SQE*1, *SQE2*, *GlOSC*1, and *GlOSC2* was extremely high in the leaf, whereas *GlOSC5*, *HMGR3*, and *GlOSC6* were highly expressed in the stolon. The expression of *SQE3*, *SQE4*, and *GlOSC5* was highest in the rattan, followed by the stolon (Figure 3A). Genes in the triterpene biosynthesis pathway were co-expressed; the expressions of *SS*, *FPS*, *HMGR2*, *SQE1*, *SQE2*, *GlOSC1*, and *GlOSC2* in the leaf were highly correlated and therefore the leaf is a possible tissue for the biosynthesis of triterpene skeletons. Real-time quantitative polymerase chain reaction (PCR) analysis was performed to uncover the expression of candidate genes in triterpenoid biosynthesis. The expression pattern of other 14 genes was similar to those in the transcriptome data, except *HMGR5* and *GlOSC4* (Figure 3B).

### Phylogenetic Analysis of Candidate Oxidosqualene Cyclase Involved in Gypenoside Biosynthesis

While *OSC*s are the key enzymes that determine the skeleton diversity of triterpenes (Phillips et al., 2006). Seven candidate *OSC*s were obtained and named *GlOSC1* to *GlOSC7*; the number of amino acids ranged from 777 to 583. The results of the alignment analysis showed that all candidates contained a DCTAE motif that is highly conserved in *OSC*s families and is responsible for initiating the cyclization reaction (Abe and Prestwich, 1995; Ito et al., 2013). *GlOSC1*, *GlOSC5*, and *GlOSC7* had conserved Y257, T364, D474, and E556, whereas four others *OSC*s had conserved H257, C364, T474, and D556. These residues were found to differentiate the steroidal-type triterpene synthases from the non-steroidal-type (Corey et al., 1997; Forestier et al., 2019). In this case, *GlOSC1*, *GlOSC5*, and *GlOSC7* were the most likely *OSC*s to cyclize oxidosqualene through reaction with the dammarenyl cation (Supplementary Figure S2).

**FIGURE 4:**
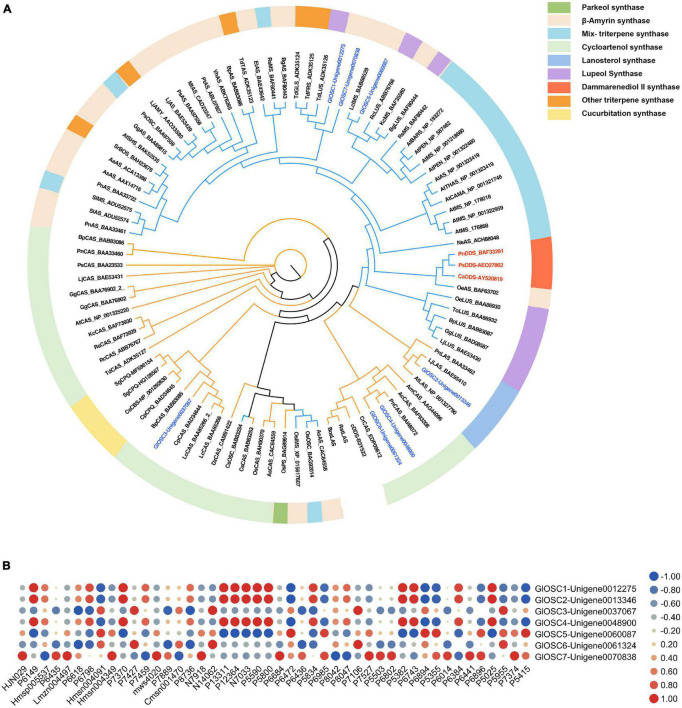
**(A)** Phylogenetic analyses of the *GlOSC*s from *G. longipes* and characterized OSCs from other plants. Their catalytic functions were classified in colors, green: parkeol synthase, light orange: β-amyrin synthase, light blue: Mix-triterpene synthase, light green: cycloartenol synthase, purple: lupeol synthase, red: dammarenediol synthase, orange: triterpene synthase with other functions, light yellow: cucurbitadienol synthase. **(B)** Pearson correlation analysis between expression level of *GlOSC*s and content level of triterpenes, the red cycle presented positive correlation, the blue cycle presented negative correlation, and size of cycle presented the degree of correlation.

The phylogenetic relationship between *GlOSC*s and known *OSC*s from plants was analyzed to support further prediction of the function of these *OSC*s. Two exogenous *OSC*s (*RatLAS* and *BosLAS*) were added for the construction of phylogenetic relationships (Figure 4A). Based on the different catalyzed cyclization reaction formation, the sequences were divided into two groups. The first group (*GlOSC2, GlOSC3, GlOSC4*, and *GlOSC5*) contained *CAS* (cycloartenol synthase), *LAS* (lanosterol synthase), *CbQ* (cucurbitadienol synthase), and *PS* (parkeol synthase), which were able to fold 2,3-oxidosqualene into the chair-boat-chair conformation through the protosteryl cation when initiating the cyclization reaction. The second group (*GlOSC5*, *GlOSC1*, and *GlOSC7*) contained *βAS* (β-amyrin synthase), *DDS* (dammarenediol II synthase), *LUS* (lupeol synthase), *MS* (mix-triterpene synthase), these were capable of folding substrates into chair–chair–chair (CCC) conformation through dammarenyl cation (Phillips et al., 2006; Thimmappa et al., 2014). This result was consistent with the results of the amino acid sequence comparison mentioned above. The first group was steroidal-type triterpene synthases; the second group was non-steroidal-type triterpene synthase.

Additionally, we performed Pearson correlation analysis between the candidate *OSC*s and all triterpenes using SPSS 26 (Figure 4B); the results showed that *GlOSC1, GlOSC2*, and *GlOSC4* had the same correlation pattern between gene expression and triterpene content. The expression was highly positively correlated with the content of P7370, P1331, P12364, P5590, and P5800, and negatively correlated with the content of P6472. The expression of *GlOSC5* was negatively correlated with the content of P1331, P12364, P5590, and P5800, however, positively correlated with the content of P6472 (Supplementary Table S3). The expression of *GlOSC3* and *GlOSC6* was negatively correlated with the content of P6618, but positively correlated with the content of N14062 and P7106. The correlation pattern between *GlOSC7* expression and the content of triterpenes is different from others; the expression of *GlOSC7* is positively correlated with the content of Lmzn004497, msw4020, and N7918, but negatively correlated with the content of P8042. All correlations mentioned above were under the conditions of a Pearson correlation coefficient (PCC) of ≥0.9 and a *p*-value of ≤0.05. These results suggest that *GlOSC1*, *GlOSC2*, and *GlOSC4* are likely connected to the biosynthesis of triterpene skeletons in the leaf.

### Candidate Cytochrome P450 Monooxygenase Involved in Gypenoside Biosynthesis

**FIGURE 5:**
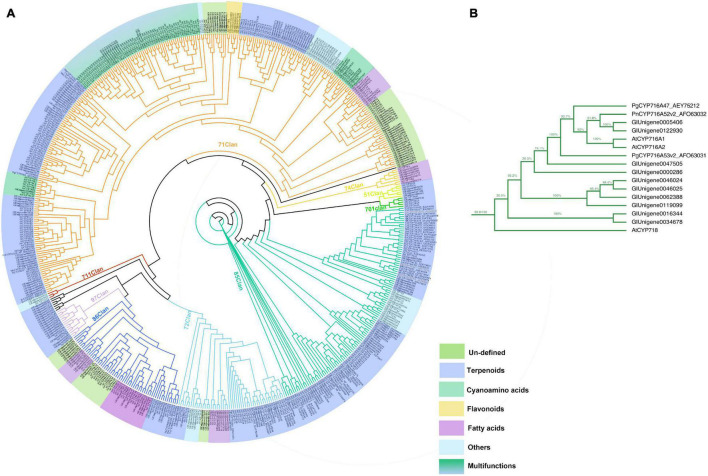
**(A)** Phylogenetic analyses of the *GlCYP*s from *G. longipes* and other reference CYPs, their catalytic functions were classified in colors, blue part related to terpenoids modification, green part related to cyanoamino acid modification, yellow part related to flavonoids modification, light blue part might have other functions, lawngreen part were unidentified. **(B)** Local zoom of Figure 5A. The phylogenetic analysis of several *GlCYP*s and PgCYP716As.

As the third largest family of plant genes, *CYP*s play an essential role in the formation and evolution of metabolism in plants (Nelson and Reichhart, 2011). *CYP*s are crucial enzymes in natural product biosynthesis and metabolism in plants; they catalyze a wide range of reactions, including hydroxylation, decarboxylation, epoxidation, *C–C* bond formation, *N*- and *O*-demethylation, and other oxidizing reactions (Jeffreys et al., 2018; Li et al., 2020). In terpenoid biosynthesis, *CYP*s determine the structural diversity and bioactivity of terpenoids upstream, with more than 97% of terpenoids modified under catalysis by *CYP*s (Guo et al., 2016; Xiao et al., 2018). In this study, 250 *CYP*s were identified by HMMER 3.0. The phylogenic tree was constructed with 250 candidate *CYP*s, 74 reference *CYPs*, and 245 *AtCYP*s by *iqtree* (Figure 5A). To date, 11 clans of plant *CYP*s have been found, and the *CYP*s of *G. longipes* are categorized into 10 clans. Seven unigenes between clan 97 and clan 711 and one unigene between clan 74 and clan 71 did not belong to any clans. Clan 71 contains the highest number of *CYP* unigenes involved in gypenoside biosynthesis. *PgCYP716A47* and *PgCYP716A53v2* are important enzymes that catalyze dammarenediol to protopanaxadiol and protopanaxadiol to protopanaxatriol, respectively (Figure 5B; Han et al., 2011, 2012). According to the results of the phylogenetic analysis, Unigene0005406 and Unigene0122930 had 51.8% identity to *PgCYP716A53v2*; thus, Unigene0005406 and Unigene0122930 are potential protopanaxadiol synthases in *G. longipes*.

### Integrated Transcriptome and Metabolite Analysis

**FIGURE 6:**
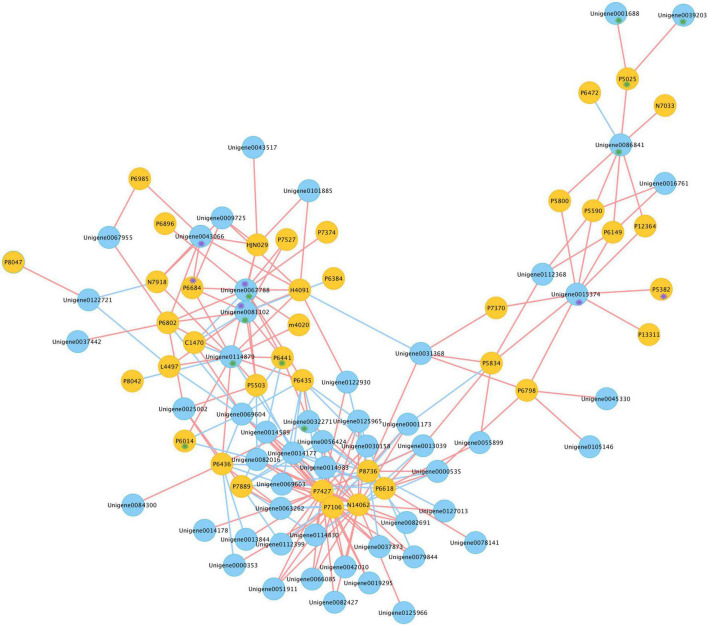
Co-expression network of candidates *CYP*s and triterpenes in *G. longipes.* Blue nodes represented genes, yellow nodes represented triterpenes, red edges presented positive correlation, blue edges presented negative correlation. Compounds marked with purple * had oxidization at C-19, unigene marked with purple * possibly catalyzed oxidization at C-19; compounds marked in green * had hydroxylation at C-2, unigene marked with green * possibly catalyzed hydroxylation at C-2.

For a better understanding of the association between genes and gypenosides, the integrated transcriptome and metabolite data were analyzed by applying a correlation analysis, and the network was constructed. Pearson correlation analysis was conducted between the expression of 209 *CYP*s and the content of 46 triterpenes using SPSS 26; only correlation pairs satisfying the conditions of PCC ≥ 0.8 and p-value ≤ 0.05 were selected. In total, 214 pairs were identified and visualized by Cytoscape (Figure 6 and Supplementary Table S3). In the network, 91 nodes were connected by 214 edges. These 91 nodes included 38 triterpenes and 53 unigenes; here, 60 pairs were negatively correlated and 154 pairs were positively correlated. From the metabolism data, P6441 (gymnemaside III), P6014 (gypenoside LVI), and P5025 (gypenoside LXI) were all hydroxylated at the C-2 position. The results of the correlation analysis showed that the content of P6441 was positively correlated with the expression of Unigene0032271 (PCC = 0.818), Unigene0067788 (PCC = 0.908), and Unigene0081102 (PCC = 0.892); the content of P6014 was positively correlated with the expression of Unigene0114879; and the content of P5025 was positively correlated with the expression of Unigene0001688 (PCC = 0.803), Unigene0039203 (PCC = 0.801), and Unigene0086841 (PCC = 0.885). Thus, these unigenes are likely to catalyze the hydroxylation at the C-2 position. In addition, P6684 [(23S)-21-*O*-ethyl-3,20,21-trihydroxy-19-oxo-21,23-epoxydammar-24-ene-3-*O*-rhamnopyranosyl-xylopyranosyl-arabinopyranoside*] and P5382 (gypenoside XLVIII*) with an aldehyde group at the C-19 position, were also in a distinct position from the gypenosides. The content of P6684 was positively correlated with the expression of Unigene0043066 (PCC = 0.812), Unigene0067788 (PCC = 0.937), and Unigene0081102 (PCC = 0.926); the content of P5382 was positively correlated with the expression of Unigene0015374 (PCC = 0.818) (Supplementary Table S2). Therefore, these three unigenes were highly likely to be related to the oxidation at the C-19 position.

### Heterologous Recombination and Protein Expression in Yeast

**FIGURE 7:**
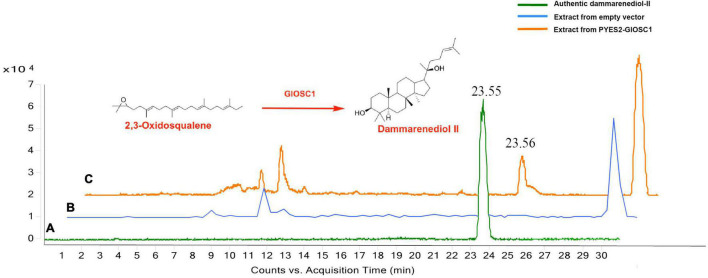
Three chromatograms of the analysis: **(A)** authentic dammarenediol II, **(B)** the extracts of the yeast with empty vector, and **(C)** dammarenediol II biosynthesized by *GlOSC1* in yeast.

The full-length open reading frame of *GlOSC1* was successfully cloned from *G. longipes* cDNA and inserted in the pYES2 expression vector under the control of the GAL1 promoter. The transgene vectors were transformed into *Saccharomyces cerevisiae* BY4742 by addition of galactose. The final product of the yeast extracts was analyzed by LC chromatogram and liquid chromatography–mass spectrometry (LC–MS) after incubation for 24 h. The results showed that the extract from transgenic yeast expressing *GlOSC1* appeared a peak at 23.56 min, which matched the retention time of the dammarenediol-II standard peak at 23.55 min. In contrast, the LC–MS of extracts from the empty vector showed no dammarenediol-II product (Figure 7). In addition, the LC–MS was performed to verify the molecular weight of the dammarenediol-II product. The spectrum of *GlOSC1* transgenic yeast showed LC/APCIMS fragmentation ion values of the dammarenediol II product at m/z 427 [M+H–H_2_O]^+^, m/z 409 [M+H–2H_2_O]^+^, m/z 219, and m/z 191, (Supplementary Figures S3, S4) which were consistent with dammarenediol-II standard previously described (Han et al., 2006). The peak at 23.56 min from the extract of transgenic yeast *GlOSC1* also had the same LC/APCIMS fragmentation ion values as those in authentic dammarenediol-II. These data indicated that the Unigene0012275 (*GlOSC1*) could catalyze 2,3-oxidosqualene to produce dammarenediol-II via cyclization.

## Discussion

Triterpenoids are not only crucial compounds for plant development and growth, but are also important sources of drugs and antibiotic for humans and other creatures (Haralampidis et al., 2002). Owing to their various biological properties, researchers are interested in their molecular structures. Currently, more than 100 distinct skeletons of triterpenoids are found in plants (Xu et al., 2004). *OSC*s are the key enzymes that determine the diversity of triterpene skeletons (Phillips et al., 2006; Cardenas et al., 2019).

Previous transcriptome studies showed that *FPS*, *SS*, *SE*, and *βAS* were highly expressed in the leaf of *G. pentaphyllum* (Chen et al., 2016; Liang et al., 2019; Xu et al., 2020). In this study, based on gene expression profiling, the expression patterns of upstream genes (*SS*, *FPS*, *HMGR2*, *SQE1*, *SQE2*, *OSC1*, *OSC2*, and *OSC4*) in the biosynthesis of gypenosides were highly correlated in the leaf. This result indicated that the leaf was the main tissue for biosynthesis of terpene skeletons in *G. pentaphyllum*. Meanwhile, the metabolism profile indicated that the content of 17 triterpenes and 20 triterpenes was relatively high in the leaf and the stolon, respectively. In addition, the content of only nine triterpenes was high in the rattan; thus, the leaf and stolon may be the main tissues for biosynthesis and modification of triterpene skeletons and the accumulation of gypenosides.

Based on their functions, the *OSC*s can be classified as steroidal synthases or non-steroidal synthase (Thimmappa et al., 2014). In this study, seven *OSC*s were obtained from the *G. longipes* transcriptome; according to the phylogenetic analysis, all candidate *OSC*s had a conserved DCTAE motif that indicated triterpene synthase activity. Unigene0012275 (*GlOSC1*), Unigene0060087 (*GlOSC5*), and Unigene0070838 (*GlOSC7*) contained conserved residues Y257, T364, D474, and E556, which belong to non-steroidal-type triterpene synthases (Ito et al., 2013). Since 2006, *PgDDS* was first identified from *P. ginseng* (Tansakul et al., 2006), and *PqDS*, *CaDDS*, and *PnDS* were subsequently identified (Kim et al., 2009; Niu et al., 2014; Wang et al., 2014). Candidate *OSC*s in this research shared low homology with these four *DDS*s. A multi-functional triterpene synthase *LsOSC1* was reported that could catalyze the formation of dammarenediol-II in *Lactuca sativa*. *TcOSC1* and *TcOSC2* were also identified in *Taraxacum coreanum* (Han et al., 2019; Choi et al., 2020). *GlOSC1* showed high homology with *LsOSC1*, *TcOSC1*, and *TcOSC2*, and the results of the functional expression experiment proved that *GlOSC1* had the ability to catalyze the formation of dammarenediol-II; this was the first OSC reported in the *Gynostemma* genus.

To date, 328 dammarane-type saponins from Gynostemma plants have been reported (Nguyen et al., 2021). The downstream *CYP*s and *UGT*s genes were also determined to affect the diversity of gypenosides. During terpenoid biosynthesis processes in plants, more than 97% *CYP*s catalyzed the structural diversity of terpenoids via oxygenation reactions (Guo et al., 2016). A previous study showed that 25% of gypenosides shared similar structures to ginsenosides; in the case of panaxadiol and 2α-hydroxypanaxadiol, this was the basic structure of gypenosides (Nagai et al., 1976; Subramaniyam et al., 2011). In this study, *GlCYPs* were categorized according to their catalytic functions in metabolism (Liu et al., 2020). The results identified a total of 123 *CYP*s that could possibly catalyze the modification of terpenoids: 7 *CYP*s may be related to the catalysis of cyanoamino acid, 5 *CYP*s might catalyze the modification of flavonoids, 29 *CYP*s might be related to the catalysis of fatty acids, 28 *CYP*s may have other catalytic functions, and 11 *CYP*s might have multiple functions when catalyzing terpenoids in plants. The catalytic function of 47 *CYP*s are still unidentified.

The homologous gene of PgCYP716A47 that catalyzed dammarenediol-II to panaxadiol in *G. longipes* was the very first step in gypenoside biosynthesis. According to the phylogenetic analysis, Unigene0005406 and Unigene0122930 had higher homology with *PgCYP716A47*; thus, these two unigenes were responsible for catalyzation of the hydroxylation at C-12. Hydroxylation at C-2α and oxidization at C-19 are also important sites for gypenoside diversity. The integrated analysis of transcriptome and metabolism was performed to narrow down possible candidate genes that catalyzed the C-12 and C-19 positions. According to the co-expression network describing triterpenes content and gene expression, Unigene0032271, Unigene0067788, Unigene0081102, Unigene0114879, Unigene0001688, Unigene00392303, and Unigene0086841 were in the pool of candidate genes that could catalyze hydroxylation at C-2 position, whereas Unigene0043066, Unigene0067788, Unigene0081102, and Unigene0015374 were classified in the candidate gene pool for the catalysis of oxidization at C-19.

In the past decade, *G. pentaphyllum* has served as an alternate candidate for *Panax genus* to fulfill the market demands for these types of compounds. Recently, the noteworthy pharmacological effects of gypenosides have opened a brand-new market in China, resulting in increasing demand for gypenosides (Su et al., 2021). However, gypenoside biosynthesis and heterologous expression is rarely studied compared with ginsenosides. In this study, the key *OSC* and *CYP* enzymes involved in gypenoside biosynthesis were illustrated and an integrated transcriptome and metabolic analysis was conducted for gypenosides. A series of genes that participated in gypenoside biosynthesis was located, offering significant input to the further characterization of the molecular mechanisms of dammarenediol-type gypenoside biosynthesis in *G. pentaphyllum*. Moreover, the first *GlOSC1* to catalyze the formation of dammarenediol-II in the *Gynostemma* genus was found. In summary, comprehensive information on the transcriptional regulation and metabolic content in *G. pentaphyllum* has been discovered, helping to advance the study of dammarenediol-type saponin biosynthesis and creating opportunities to engineer microorganisms for the *de novo* production of valuable gypenosides.

## Materials and Methods

### Plant Materials

Wild *G. longipes* plants were collected from Ankang county, Shaanxi province (Supplementary Figure S5). The leaf, rattan, and stolon were collected separately, frozen in liquid nitrogen, and stored at −80°C. Three replicates of each tissue were collected and divided into two parts for metabolism and transcriptome analysis.

### Transcriptome Sequencing and Functional Annotation

RNA from the leaf, rattan, and stolon was extracted and reversed transcribed into cDNA. A Poly(A) tail was added after purification of the cDNA fragment and Illumina sequencing adapter ligation was applied. The final sequences were sequenced using Illumina Hiseq™ 4000 by Gene Denovo Biotechnology Co. (Guangzhou, China). Three biological repetitions of each tissue sample were processed. The raw reads obtained from sequencing machines; after filtering out low-quality reads (those containing more than 40% of low-quality (*Q*-value ≤10) bases), the clean reads were assembled by Trinity using default parameters (Grabherr et al., 2011).

For the functional annotation of unigenes, the BLASTx program^2^ was used with an E-value threshold of 1e-5 in the NCBI non-redundant protein (Nr) database^3^, the Swiss-Prot protein database^4^ the Kyoto Encyclopedia of Gens and Genomes (KEGG) database^5^, and the COG/KOG database^6^. Based on the Nr annotation results, the GO annotation was analyzed by Blast2GO software. Then, the functional classification of unigenes was performed using WEGO software (Ashburner et al., 2000; Ye et al., 2006). The Nr, Swiss-Prot, KEGG, and COG/KOG databases, BLAST and ESTscan programs were used for protein coding sequence (CDS) prediction (Iseli et al., 1999).

### Phylogenetic Analysis

The phylogenetic analysis of *GlOSCs* and *GlCYPs* was performed on the deduced amino acid sequences to avoid other referential sequences from plants and two exogeneous sequences from animals. All deduced acid sequences were aligned with Clustal X under default parameters (Jiang et al., 2014). The preferred IQ-TREE version 1.6.12 on alignments was used to calculate the best model according to the software instructions, as described by Kalyaanamoorthy et al. (2017). From the results of the modelfinder, LG+I+G4 was the most suitable model for the construction of phylogenetic trees for this study. Figtree version 1.4.4 was used for data visualization.

### Metabolite Extraction, Multiple Reaction Monitoring, and Parameter Setting

Multiple reaction monitoring (MRM) was performed by Metware Biotechnology Co. Lit (Wuhan, China). After the addition of zirconia beads, samples of the leaf, rattan, and stolon from *G. longipes* were processed, each tissues had three biological duplicates. The samples were freeze-dried and ground into a fine powder by a mixing mill (MM400, Restch, Haan, Germany). Then, 100 mg of sample powder was dissolved in 1 mL of 70% methanol. The sample was held at 4°C overnight and then centrifuged for 10 min at 12,000 × *g* to obtain the filtrate. The extracts were analyzed by UPLC-ESI-MS/MS (UPLC, SHIMADZU Nexera X2^7^; MS, Applied Biosystems 4500 Q TRAP^8^). The following analysis conditions were used: UPLC column, Agilent SB-C18 (1.8 μm, 2.1 mm × 100 mm); mobile phase solvent A, water with 0.1% formic acid, solvent B acetonitrile with 0.1% formic acid; gradient program, starting at 95:5 (A:B), then at 9 min 5:95 (A:B) and held for 1 min, 95:5 (A:B) at 10 min, from 95:5 (A:B) from 11.1 to 14 min; flow rate 0.35 mL/min; temperature 40°C; and injection volume 4 μL. The effluent was connected to an ESI-triple quadrupole-linear ion trap (Q-TRAP)-MS. LIT and triple quadrupole (QQQ) scans were acquired on a triple quadrupole-linear ion trap mass spectrometer (Q TRAP), AB4500 Q TRAP UPLC–MS/MS System, equipped with an ESI Turbo Ion-Spray interface, operating in positive and negative ion mode and controlled by Analyst 1.6.3 software (AB Sciex). The ESI source operation parameters were as follows: ion source, turbo spray; source temperature 550°C; ion spray voltage (IS) 5500 V (positive ion mode)/−4500 V (negative ion mode); ion source gas I (GSI), gas II (GSII), curtain gas (CUR) pressure of 50, 60, and 25.0 psi, respectively; collision-activated dissociation (CAD), high. Instrument tuning and mass calibration were performed with 10 and 100 μmol/L polypropylene glycol solutions in QQQ and LIT modes, respectively. QQQ scans were acquired as MRM experiments with the collision gas (nitrogen) set to medium. The decluttering potential (DP) and collision energy (CE) for individual MRM transitions was performed with further DP and CE optimization. A specific set of MRM transitions was monitored for each period according to the metabolites eluted within this period. The metabolites were annotated by Metware datebase^9^).

### Analysis of Gene Expression

In the transcriptome, the expression of unigenes was calculated and normalized to RPKM (reads per kb per million reads) values by BWA program (Mortazavi et al., 2008). TBtools version 1.0 was used for gene expression profiling (Chen et al., 2020).

### Real-Time Quantitative Polymerase Chain Reaction

Six *GlOSCs* and 10 other unigenes that participated in the MVA pathway were selected for RT-qPCR analysis relative to the reference gene *ACT1*. One microgram of RNA of each sample was reverse-transcribed to cDNA by using PrimeScript™ RT Reagent kit with gDNA Eraser (Takara, Dalian, China). The specific primers were designed through online Primer3Web version 4.0.0^10^ (Supplementary Table S1). The quantitative reactions were performed in a volume of 20 μL, which comprised 1 μL of cDNA, 0.4 μL of primer1, 0.4 μL of primer2, 10 μL of 2 × Taq Pro Universal SYBR qPCR Master Mix (Vazyme, Nanjing, China) and 8.8 μL of ddH_2_O and analyzed by Quantstudio 5K Flex Real-Time polymerase chain reaction System (Applied Biosystems, Foster City, CA, United States) under the following conditions: 1 cycle of 95°C (30 s), 40 cycles of 95 (10 s) and 60°C (30 s), 1 cycle of 95°C (15 s), 60°C (60 s), and 95°C 15 s. The relative gene expression was calculated using the 2^–ΔΔCt^ method (Livak and Schmittgen, 2001).

### Statistical Analysis and Gene Metabolism Network Construction

The Pearson model was used to determine the correlation analysis between gene expression and chemical content; the analysis was performed using SPSS version 26. Correlation pairs under the conditions of PCC ≥ 0.9 and *p*-value ≤ 0.05 were selected and visualized by Cytoscape 3.8.2.

### Vector Construction and Functional Expression in *Saccharomyces cerevisiae* BY4742

The protein coding sequences (CDS) of *GlOSC1* (Unigene0012275) were obtained from *G. longipes* transcriptome. The primers for fragment PCR were designed using SnapGene. The PCR reactions were performed with Phanta Super-Fidelity DNA Polymerase (Vazyme Biotech Co., Ltd. Nanjing, China), processed in accordance with the user manual^11^. The PCR fragments were ligated into the *BamHI* site of the PYES2 vector under the control of the *GAL1* promoter by the ClonExpress II One Step Cloning Kit (Vazyme Biotech Co., Ltd., Nanjing, China) and then transferred into the mix buffer into *Escherichia coli* line DH5α (TransGen Biotech. Co., Ltd., Beijing, China) for cloning. After incubation at 37°C overnight in LB medium with ampicillin, the reconstructed plasmids were extracted by *EastPure* Hipure Plasmid Maxiprep Kit (TransGen Biotech. Co., Ltd., Beijing, China).

*GlOSC1-PYES2* and an empty vector yeast strain BY4742 (Miaoling Biotechnology Co., Ltd., Wuhan, China) were transformed by the lithium acetate method (Kushiro et al., 1998). A single clone was incubated in complete medium without uracil (CM-U) at 30°C with shaking at 220 g for 48 h; otherwise, 13 mg/L hemin and 5 g/L Tween 80 were included in CM-U. The glucose medium was replaced with galactose medium for induction for 48 h at 30°C, 220 g further incubated in 0.1 M potassium phosphate for 1 day. Finally, 50 mL of methyl alcohol was refluxed into the cells and then ultrasound treatment was applied for 30 min.

### Identification of Products by High Performance Liquid Chromatography and Liquid Chromatography–Mass Spectrometry Analysis

The yeast extracts were analyzed by UPLC-QTOF-MS performed on an Agilent UPLC time-of-flight mass spectrometer (UPLC/MS-TOF) under a fragment dissociation voltage of 135 V. A high performance liquid chromatography (HPLC) Agilent Zorbax SB-C18 column (250 mm × 4.6 mm, 5 μm) was used for chemical separation, with a column temperature of 25°C. The mobile phase solvent A was water with 0.1% formic acid, solvent B was acetonitrile, and the following linear gradient elution was applied: 0–20 min, 20%→10% A; 20–28 min; 10% A; 28–29 min, 20%→10% A; 29–31 min, 20% A; the flow rate was 0.5 mL/min, the wavelength was 210 nm, and the injection volume was 10 μL.

## Conclusion

The saponins and related genes involved biosynthesis were analyzed by the integrated analysis of the metabolome and transcriptome in the leaf, stolon, and rattan of *G. longipes*. The saponin skeletons were mainly biosynthesized in the leaf by *OSCs*, and then modified via *CYPs* and *UGTs* to form various ginsenoside and gypenosides in the stolon. The catalytic function of *GlOSC1* encoded by Unigene0012275 was heterologous expression in yeast to verify the ability of 2,3-oxidosqualene to catalyze the formation of dammarenediol-II via cyclization. The study has also provided essential information for the better utilization of *G. longipes* to produce valuable ginsenosides and gypenosides with excess substrate in a yeast cell factory through a synthetic biology strategy.

## Data Availability Statement

The datasets presented in this study can be found in online repositories. The names of the repository/repositories and accession number(s) can be found below: https://www.ncbi.nlm.nih.gov/, PRJNA784129.

## Author Contributions

BF, GZ, and SCY conceived the study. SY, LF, SYZ, QW, and WS performed the experiments. SY, LF, SYZ, and GZ the designed experiments. SY, GX, BN, LY, and XL analyzed the data. SY, SYZ, BH, and GZ drafted the manuscript. BH, GZ, and SCY reviewed and edited the manuscript. All authors contributed to the article and approved the submitted version.

## Conflict of Interest

BF was employed by Honwin Pharma (Lianghe) Co., LTD. The remaining authors declare that the research was conducted in the absence of any commercial or financial relationships that could be construed as a potential conflict of interest.

## Publisher’s Note

All claims expressed in this article are solely those of the authors and do not necessarily represent those of their affiliated organizations, or those of the publisher, the editors and the reviewers. Any product that may be evaluated in this article, or claim that may be made by its manufacturer, is not guaranteed or endorsed by the publisher.

## Footnotes

1
http://medicinalplants.ynau.edu.cn/transcriptomics/221

2
http://www.ncbi.nlm.nih.gov/BLAST/

3
http://www.ncbi.nlm.nih.gov

4
http://www.expasy.ch/sprot

5
http://www.genome.jp/kegg

6
http://www.ncbi.nlm.nih.gov/COG

7
www.shimadzu.com.cn/

8
www.appliedbiosystems.com.cn/

9
https://www.metware.cn/nxjszc12.html?questionCateId=4

10
http://www.primer3plus.com

11
http://www.vazyme.com/product/116.html

## Supplementary Material

Supplementary Figure S1 **(A)** Functional classification by KEGG. The number of genes annotated to pathways, including Cellular Processes, Environmental Information Processing, Genetic Information Processing, and Metabolism. **(B)** Venn diagram of the KEGG, SwissProt, Pfam, and GO results for the *G. longipes* transcriptome. **(C)** Functional classification by GO. The number of genes annotated into the three categories: Biological Process, Cellular Component, and Molecular Function.

Supplementary Figure S2 Comparison of *GlOSC*s amino sequences; the sequences in the yellow box are the conserved motif (DCTAE) for all *OSC*s; the sequences in the green box are the amino acid sites to distinguish steroidal synthases and non-steroidal synthases.

Supplementary Figure S3 LC–MS analysis of authentic dammarenediol-II.

Supplementary Figure S4 LC–MS analysis of products from *PYES2-GlOSC1.*

Supplementary Figure S5 Images of *G. longipes* and tissues used in this research are marked in white.

Supplementary Table S1 Primer sequences used for gene expression by qPCR.

Supplementary Table S2 Pearson correlation analysis between the levels of candidate triterpenes and the expression level of candidate genes.

Supplementary Table S3 Comparison table between index and compounds names, and their relative content.

## Abbreviations

*DDS*: dammarenediol-II synthase
MVA: mevalonate
MEP: methylerythritol phosphate
*OSC*: oxidosqualene cyclase
*CYP*: cytochrome P450 monooxygenase
*UGT*: UDP-dependent glycosyltransferases
HMGR: 3-hydroxy-3-methylglutaryl-coenzyme A reductase
*FPS*: farnesyl diphosphate synthase
*SS*: squalene synthase
*SQE*s: squalene epoxidase
*CAS*: cycloartenol synthase
*LAS*: lanosterol synthase
*CbQ*: cucurbitadienol synthase
*PS*: parkeol synthase
*β AS*: β-amyrin synthase
*LUS*: lupeol synthase
*MS*: mix-triterpene synthase
CDS: protein coding sequences
PPD: protopanaxadiol.
