# Supplementary material for: Integrated Metabolomic and Transcriptomic Analysis and Identification of Dammarenediol-II Synthase Involved in Saponin Biosynthesis in Gynostemma longipes
Source: Front Plant Sci. 2022 Mar 25;13:852377. doi: 10.3389/fpls.2022.852377 (PMC8990310; doi:10.3389/fpls.2022.852377)

**Table S1. Primer sequence used for gene expression by qPCR**

| **Primer name** | **Sequence** | **Length（bp）** |
| --- | --- | --- |
| *Glhmgr2-F*  *Glhmgr2-R* | GTGGTGCTGGTAGCGTTTTG  CCCAGCCATAGCACACTTGA | 20  20 |
| *Glhmgr3-F* | AACTCGCGTCTCAATCTGCA | 20 |
| *Glhmgr3-R* | TGCAAGAACTGAACCAGCCA | 20 |
| *Glhmgr4-F* | TCCCTCTCCATGTCGTCACT | 20 |
| *Glhmgr4-R* | CGATGTCGGTTTCGGTTTCG | 20 |
| *Glhmgr5-F* | TGGGGATGAACATGGTGTCG | 20 |
| *Glhmgr5-R* | GCCTTTTTGTCAGCGCAGAA | 20 |
| *Glss-F* | TGGCTGCTAGAAATGCGGAA | 20 |
| *Glss-R* | CTCAACGGTATCGAGGGCTC | 20 |
| *Glse1-F* | GACACCCGGTGCCTTATTGA | 20 |
| *Glse1-R* | AGGTTCCGAAGCACAACGAT | 20 |
| *Glse2-F* | ACTCTTGGCAAGGATGGACG | 20 |
| *Glse2-R* | CACTGTCCTCGAGACCCAAC | 20 |
| *Glse3-F* | AACTGGCGGAGGAATGACTG | 20 |
| *Glse3-R* | GGTGAAGCGCAAAACACCTT | 20 |
| *Glse4-F* | GGTGCACTTCTGATGGGTGA | 20 |
| *Glse4-R* | AGCATCGTCCAAATCCTGCA | 20 |
| *Glfps-F* | GGCGGGTGAAAATCTGGAGA | 20 |
| *Glfps-R* | GTTCCCACCTTTCCGATGGT | 20 |
| *Glosc1-F* | GCTGCTGATATGGTCGACGA | 20 |
| *Glosc1-R* | CACGGTCCGAGAAAGTCCAA | 20 |
| *Glosc2-F* | GGACAGGCTGAAAGAGACCC | 20 |
| *Glosc2-R* | GCGATACTCTCCAAGAGCCC | 20 |
| *Glosc3-F* | TTACCCACATCCGCTGGTTC | 20 |
| *Glosc3-R* | GCAAGGCCTTCTCCCTCAAT | 20 |
| *Glosc4-F* | CGCCTTGCAGTCGATAAAGC | 20 |
| *Glosc4-R* | GCTGCCCGTTTCAATGTGTT | 20 |
| *Glosc5-F* | ATGCGGTTTCAAGAGCGAGA | 20 |
| *Glosc5-R* | CGACCACTCGAACAGGTTGA | 20 |
| *Glosc6-F* | ATGCGGTTTCAAGAGCGAGA | 20 |
| *Glosc6-R* | CGACCACTCGAACAGGTTGA | 20 |

| Triterpenes ID | PCC | P value | PCC | P value | PCC | P value | PCC | P value | PCC | P value |  |
| --- | --- | --- | --- | --- | --- | --- | --- | --- | --- | --- | --- |
| Gene ID | P6684 | | P5382 | | P6014 | | P6441 | | P5025 | |  |
|  |  |  |  |  |  |  |  |  |  |  |  |
| Unigene0001688 |  |  |  |  |  |  |  |  | .803** | 0.009 |  |
| Unigene0039203 |  |  |  |  |  |  |  |  | .801** | 0.010 |  |
| Unigene0114879 | .889** | 0.001 |  |  | .823** | 0.006 | .899** | 0.001 |  |  |  |
| Unigene0015374 |  |  | .818** | 0.007 |  |  |  |  |  |  |  |
| Unigene0032271 |  |  |  |  |  |  | .818** | 0.007 |  |  |  |
| Unigene0043066 | .812** | 0.008 |  |  |  |  |  |  |  |  |  |
| Unigene0067788 | .937** | 0.000 |  |  |  |  | .908** | 0.001 |  |  |  |
| Unigene0081102 | .926** | 0.000 |  |  |  |  | .892** | 0.001 |  |  |  |
| Unigene0086841 |  |  |  |  |  |  |  |  | .885** | 0.002 |  |

**Table S2. Pearson Correlation analysis between contents of candidate triterpenes and expression level of candidate genes.**

**Table S3. Comparison table between index and compounds names**

| Index | Formula | Compounds | Leaf1 | Leaf2 | Leaf3 | Stolon1 | Stolon2 | Stolon3 | Rattan1 | Rattan2 | Rattan3 |
| --- | --- | --- | --- | --- | --- | --- | --- | --- | --- | --- | --- |
| HJN029 | C_53_H_90_O_23_ | Sanchirhinoside A6 | 7.22E+04 | 7.69E+04 | 8.90E+04 | 3.98E+05 | 3.46E+05 | 2.62E+05 | 4.13E+04 | 3.32E+04 | 3.26E+04 |
| P6149 | C_48_H_80_O_19_ | Notoginsenoside G | 1.64E+04 | 1.91E+04 | 1.55E+04 | 1.18E+04 | 8.09E+03 | 1.23E+04 | 8.63E+03 | 9.50E+03 | 1.02E+04 |
| Hmsp005537 | C_36_H_62_O_10_ | Notoginsenoside R9 | 2.31E+04 | 1.93E+04 | 2.18E+04 | 1.64E+04 | 1.61E+04 | 1.36E+04 | 2.13E+04 | 2.09E+04 | 1.86E+04 |
| P6435 | C_48_H_78_O_17_ | (23S)-21-O-ethyl-3,20,21-trihydroxy-19-oxo-21,23-epoxydammar-24-ene 3-O-rhamnopyranosyl-xylopyranosyl-arabinopyranoside. | 5.05E+04 | 4.80E+04 | 5.72E+04 | 7.36E+04 | 7.96E+04 | 6.95E+04 | 3.00E+04 | 3.90E+04 | 3.92E+04 |
| Lmzn004497 | C_58_H_98_O_26_ | Notoginsenoside Fc | 3.99E+04 | 3.90E+04 | 5.08E+04 | 1.01E+05 | 8.84E+04 | 1.02E+05 | 3.62E+04 | 3.89E+04 | 4.28E+04 |
| P6618 | C_48_H_82_O_19_ | Gypenoside XLIV; Gypenoside XLVI | 3.96E+04 | 4.02E+04 | 3.68E+04 | 4.02E+04 | 4.16E+04 | 3.31E+04 | 2.27E+04 | 2.44E+04 | 2.18E+04 |
| P6798 | C_36_H_62_O_9_ | Gypenoside LXXⅥ | 3.55E+05 | 3.71E+05 | 3.93E+05 | 3.73E+05 | 3.50E+05 | 2.95E+05 | 2.98E+05 | 2.91E+05 | 3.07E+05 |
| Hmsn004091 | C_54_H_92_O_23_ | Notoginsenoside Rb1 | 1.22E+05 | 1.22E+05 | 1.21E+05 | 1.99E+05 | 1.87E+05 | 2.46E+05 | 2.00E+05 | 2.45E+05 | 3.33E+05 |
| Hmsn004349 | C_53_H_90_O_22_ | Notoginsenoside L | 1.51E+03 | 7.09E+03 | 5.17E+03 | 8.90E+04 | 8.13E+04 | 7.06E+04 | 1.15E+04 | 1.94E+04 | 1.42E+04 |
| P7370 | C_46_H_74_O_16_ | 23-H-3,20-dihydroxy-19-oxo-21,23-epoxydammar-24-ene-3-O-rhamnopyranosyl-xylopyranosyl-arabinopyranoside | 6.94E+04 | 7.06E+04 | 8.83E+04 | 2.09E+04 | 1.79E+04 | 1.74E+04 | 1.39E+04 | 1.56E+04 | 1.63E+04 |
| P7427 | C_42_H_70_O_15_ | Gypenoside GD1 | 1.50E+04 | 1.44E+04 | 1.34E+04 | 9.00E+00 | 9.00E+00 | 9.00E+00 | 5.42E+04 | 5.98E+04 | 5.56E+04 |
| P7459 | C_47_H_80_O_17_ | Ginsenoside Rd2 | 3.85E+03 | 4.75E+03 | 5.62E+03 | 1.87E+03 | 1.94E+03 | 2.49E+03 | 4.73E+03 | 3.51E+03 | 3.70E+03 |
| mws4020 | C_53_H_90_O_22_ | Ginsenoside Rb2 | 9.00E+00 | 9.00E+00 | 9.00E+00 | 8.79E+03 | 1.03E+04 | 1.04E+04 | 9.00E+00 | 9.00E+00 | 9.00E+00 |
| P7889 | C_41_H_68_O_12_ | 19-oxo-20(S)-hydroxydammar-24-ene-3-O-β-glucopyranosyl-arabinopyranoside | 1.74E+04 | 1.13E+04 | 1.57E+04 | 1.63E+04 | 1.90E+04 | 1.60E+04 | 9.98E+03 | 7.26E+03 | 8.72E+03 |
| Cmsn001470 | C_44_H_72_O_15_ | O-acetyl-O-glucoside-Ginsenoside Rh7 | 1.99E+04 | 2.69E+04 | 2.37E+04 | 6.30E+03 | 8.82E+03 | 5.93E+03 | 3.90E+04 | 2.20E+04 | 3.13E+04 |
| P8736 | C_48_H_82_O_21_ | ( 2α, 3β, 12β, 23E)-20-(β-D-Glucopyranosyloxy)-25-hydroperoxy-2,12-dihydroxydammar-23-en-3-yl-2-O-β-D-glucopyranosyl-β-D-glucopyranoside | 1.05E+04 | 1.18E+04 | 1.14E+04 | 8.48E+03 | 8.66E+03 | 9.03E+03 | 9.00E+00 | 9.00E+00 | 9.00E+00 |
| N7918 | C_42_H_70_O_14_ | Gypenoside XXXIII | 7.39E+03 | 6.55E+03 | 7.48E+03 | 4.11E+04 | 3.60E+04 | 3.71E+04 | 1.02E+04 | 8.70E+03 | 9.47E+03 |
| N14062 | C_30_H_48_O_3_ | (23S)-3β-hydroxydammar-21-oic acid 21,23-lactone | 7.47E+03 | 9.10E+03 | 6.10E+03 | 9.86E+03 | 1.48E+04 | 1.35E+04 | 4.51E+04 | 3.93E+04 | 3.82E+04 |
| P13311 | C_46_H_74_O_17_ | Gypenoside A | 1.28E+06 | 1.32E+06 | 1.45E+06 | 2.83E+03 | 4.48E+03 | 3.55E+03 | 5.57E+04 | 6.64E+04 | 7.36E+04 |
| P12364 | C_41_H_70_O_13_ | Gypenoside XXI | 6.06E+05 | 6.10E+05 | 5.98E+05 | 3.50E+03 | 3.32E+03 | 3.12E+03 | 1.84E+04 | 1.85E+04 | 1.98E+04 |
| N7033 | C_43_H_72_O_15_ | 23-O-Acetyl-3,12,23,24-tetrahydroxy-20,25-epoxydammarane-3-O-xylopyranosyl-glucopyranoside | 4.34E+04 | 4.50E+04 | 4.29E+04 | 4.52E+03 | 4.83E+03 | 6.15E+03 | 1.21E+04 | 7.56E+03 | 1.13E+04 |
| P5590 | C_47_H_76_O_17_ | (20S)-3-β-20,23-trihydroxydammaar-24-en-21-oic acid-21, 23-lactone | 3.35E+05 | 3.26E+05 | 2.93E+05 | 1.26E+05 | 1.30E+05 | 9.74E+04 | 1.14E+05 | 1.39E+05 | 1.17E+05 |
| P5800 | C_47_H_76_O_17_ | (20R)-3β-20,23-trihydroxydammaar-24-en-21-oic acid-21,23-lactone* | 3.42E+05 | 3.52E+05 | 3.49E+05 | 1.42E+05 | 1.22E+05 | 1.22E+05 | 1.38E+05 | 1.38E+05 | 1.21E+05 |
| P6684 | C_48_H_78_O_17_ | (23S)-21-O-ethyl-3,20,21-trihydroxy-19-oxo-21,23-epoxydammar-24-ene 3-O-rhamnopyranosyl-xylopyranosyl-arabinopyranoside* | 1.75E+05 | 1.76E+05 | 1.90E+05 | 3.55E+05 | 3.16E+05 | 3.28E+05 | 1.53E+05 | 1.45E+05 | 1.45E+05 |
| P6472 | C_47_H_78_O_18_ | Gypenoside XXV | 9.81E+02 | 1.15E+03 | 1.35E+03 | 4.58E+03 | 4.96E+03 | 4.52E+03 | 5.53E+03 | 3.46E+03 | 5.10E+03 |
| P6436 | C_48_H_80_O_18_ | 3β,20S-dihydroxydammar-24-ene-21-carboxylic acid-3-O-rhamnopyranosyl-glucopyranosyl-β-D-glucopyranoside | 1.44E+04 | 1.32E+04 | 1.15E+04 | 1.68E+04 | 1.67E+04 | 1.46E+04 | 7.02E+03 | 9.76E+03 | 5.63E+03 |
| P5834 | C_47_H_80_O_19_ | 3β,12β,23S,24R-tetrahydroxy-20s,25-epoxydammarane 3-O-glucopyrano-xylopyranosyl-glucopyranoside | 1.79E+04 | 1.89E+04 | 1.83E+04 | 1.13E+04 | 1.25E+04 | 1.18E+04 | 7.32E+03 | 7.00E+03 | 5.71E+03 |
| P6985 | C_51_H_80_O_19_ | (3,20,23)-3-[O-4-O-acetyl-6-deoxy-mannopyranosyl-O-xylopopyranosyl]-6-O-acetyl-glucopyranosyl] oxy]-20,23-dihydroxydammar-24-en-21-oic acid 21,23-lactone* | 4.63E+03 | 4.52E+03 | 4.53E+03 | 1.35E+04 | 6.53E+03 | 1.06E+04 | 7.80E+03 | 7.40E+03 | 7.01E+03 |
| P8042 | C_51_H_80_O_19_ | (3,20,23)-3-[O-4-O-acetyl-6-deoxy-mannopyranosyl-O-xylopopyranosyl]-6-O-acetyl-glucopyranosyl] oxy]-20,23-dihydroxydammar-24-en-21-oic acid 21,23-lactone | 6.90E+04 | 6.81E+04 | 6.53E+04 | 3.51E+04 | 4.04E+04 | 3.61E+04 | 7.16E+04 | 6.68E+04 | 6.50E+04 |
| P8047 | C_51_H_84_O_21_ | m-Gin-Rd* | 1.70E+04 | 1.86E+04 | 1.79E+04 | 3.99E+03 | 3.89E+03 | 4.32E+03 | 1.44E+04 | 1.47E+04 | 1.36E+04 |
| P7106 | C_51_H_84_O_21_ | m-Gin-Rd | 4.82E+03 | 5.95E+03 | 4.87E+03 | 6.22E+03 | 4.06E+03 | 4.49E+03 | 9.99E+03 | 9.47E+03 | 1.04E+04 |
| P7527 | C_52_H_88_O_21_ | Gypenoside 16（XV） | 9.00E+00 | 9.00E+00 | 9.00E+00 | 2.87E+04 | 2.37E+04 | 2.65E+04 | 4.54E+03 | 4.27E+03 | 4.18E+03 |
| P5503 | C_53_H_88_O_22_ | Gypenoside XLVIII | 4.28E+04 | 3.98E+04 | 4.07E+04 | 8.42E+04 | 7.43E+04 | 8.26E+04 | 1.50E+04 | 1.82E+04 | 2.13E+04 |
| P6802 | C_53_H_88_O_22_ | Gypenoside VN2 | 2.28E+05 | 2.15E+05 | 2.28E+05 | 4.47E+05 | 3.59E+05 | 3.97E+05 | 1.33E+05 | 1.42E+05 | 1.34E+05 |
| P5382 | C_53_H_88_O_22_ | Gypenoside XLVIII* | 8.21E+04 | 7.78E+04 | 9.61E+04 | 2.12E+04 | 2.02E+04 | 1.64E+04 | 1.12E+04 | 1.74E+04 | 1.39E+04 |
| P6743 | C_53_H_90_O_22_ | Ginsenoside Rb3 | 2.18E+05 | 2.41E+05 | 2.61E+05 | 5.97E+04 | 4.94E+04 | 4.64E+04 | 1.00E+05 | 9.94E+04 | 9.17E+04 |
| P6894 | C_54_H_90_O_22_ | Gypenoside LXIX | 8.92E+03 | 8.41E+03 | 8.72E+03 | 9.03E+03 | 1.25E+04 | 1.05E+04 | 1.07E+04 | 9.88E+03 | 1.24E+04 |
| P5355 | C_53_H_90_O_23_ | Gypenoside XXII | 9.00E+00 | 9.00E+00 | 9.00E+00 | 4.41E+04 | 3.33E+04 | 3.34E+04 | 2.55E+04 | 2.04E+04 | 2.45E+04 |
| P6014 | C_53_H_90_O_23_ | Gypenoside LVI | 1.32E+04 | 1.12E+04 | 1.34E+04 | 1.63E+04 | 1.44E+04 | 1.50E+04 | 9.50E+03 | 1.31E+04 | 9.38E+03 |
| P6384 | C_53_H_90_O_23_ | Gypenoside LVI* | 2.27E+03 | 2.72E+03 | 2.05E+03 | 9.00E+00 | 9.00E+00 | 9.00E+00 | 8.49E+02 | 1.37E+03 | 1.24E+03 |
| P6441 | C_54_H_90_O_23_ | （Gymnemaside III）2-Hydroxy-3-[(2-O-beta-D-glucopyranosyl-beta-D-glucopyranosyl)oxy]-20-[(6-O-alpha-L-rhamnopyranosyl-beta-D-glucopyranosyl)oxy]dammar-24-en-12-one | 8.12E+05 | 8.70E+05 | 8.73E+05 | 1.61E+06 | 1.66E+06 | 1.64E+06 | 6.07E+05 | 5.90E+05 | 4.88E+05 |
| P6896 | C_54_H_92_O_23_ | Gypenoside Rd | 1.68E+05 | 1.39E+05 | 1.65E+05 | 3.21E+05 | 3.11E+05 | 2.93E+05 | 2.10E+05 | 2.05E+05 | 2.46E+05 |
| P5025 | C_53_H_90_O_24_ | Gypenoside LXI | 6.24E+05 | 4.65E+05 | 4.67E+05 | 3.10E+05 | 3.34E+05 | 3.03E+05 | 3.16E+05 | 3.12E+05 | 3.89E+05 |
| P5955 | C_53_H_90_O_24_ | Gypenoside LXVIII | 1.07E+05 | 9.26E+04 | 7.28E+04 | 1.10E+05 | 7.50E+04 | 1.17E+05 | 1.20E+05 | 1.20E+05 | 1.05E+05 |
| P7374 | C_55_H_92_O_23_ | Gypenoside VN1 | 3.91E+04 | 3.62E+04 | 3.72E+04 | 1.58E+05 | 1.69E+05 | 1.87E+05 | 6.55E+04 | 5.21E+04 | 5.43E+04 |
| P5415 | C_54_H_92_O_24_ | Notoginsenoside A | 9.00E+00 | 9.00E+00 | 9.00E+00 | 3.29E+04 | 3.11E+04 | 3.68E+04 | 2.20E+04 | 2.71E+04 | 2.65E+04 |

Supplementary Figure S1


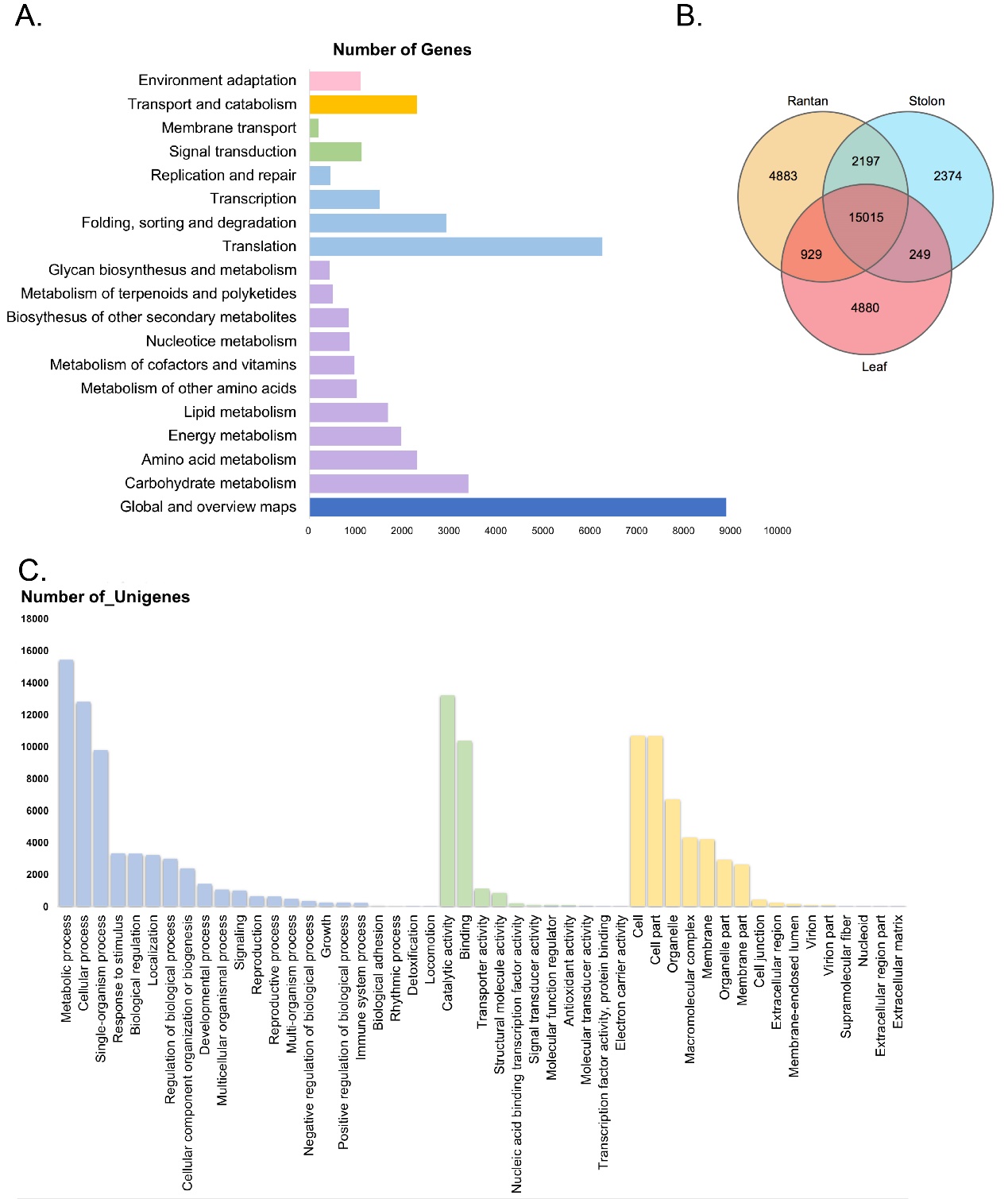


Supplementary Figure S2


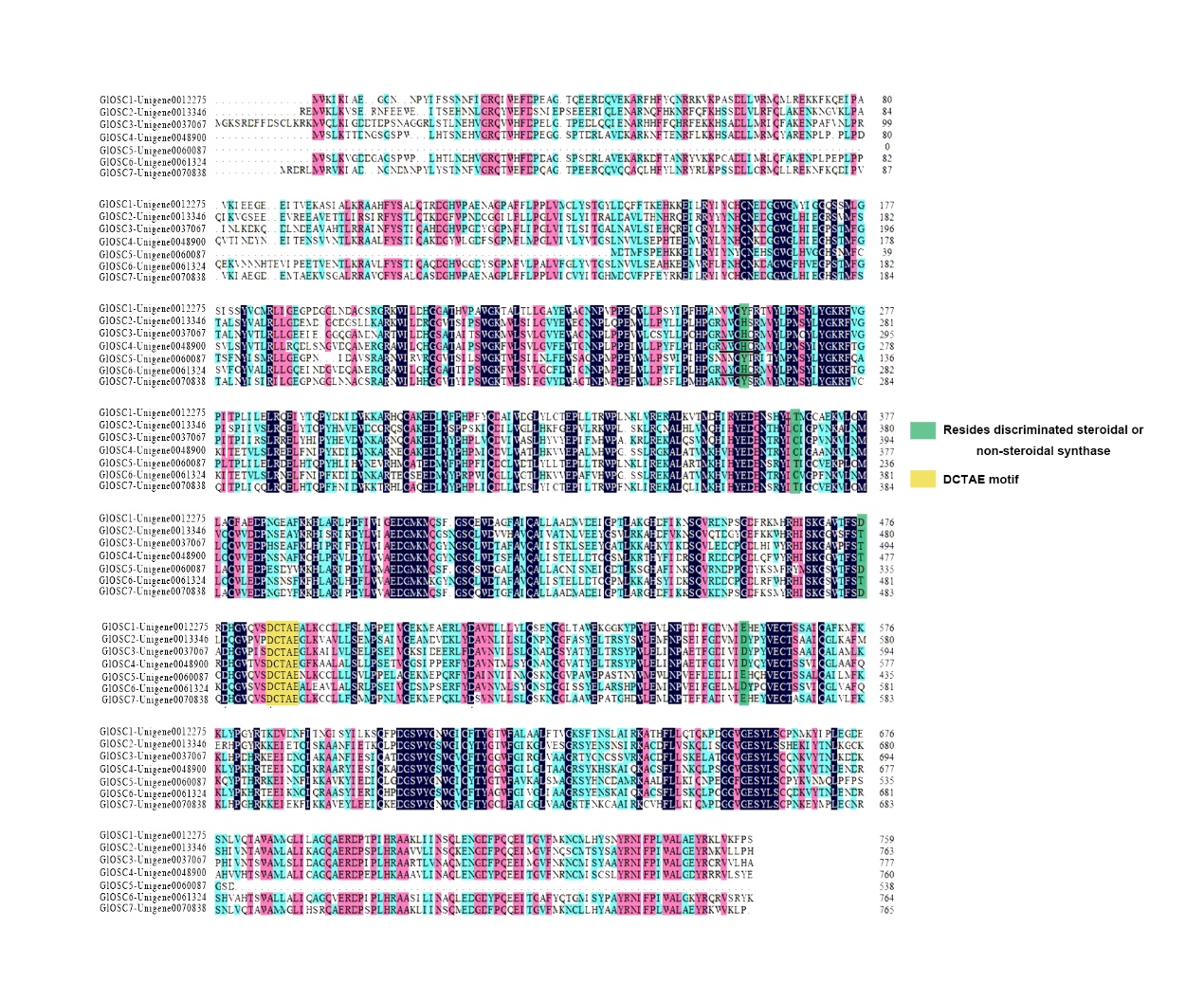


Supplementary Figure S3


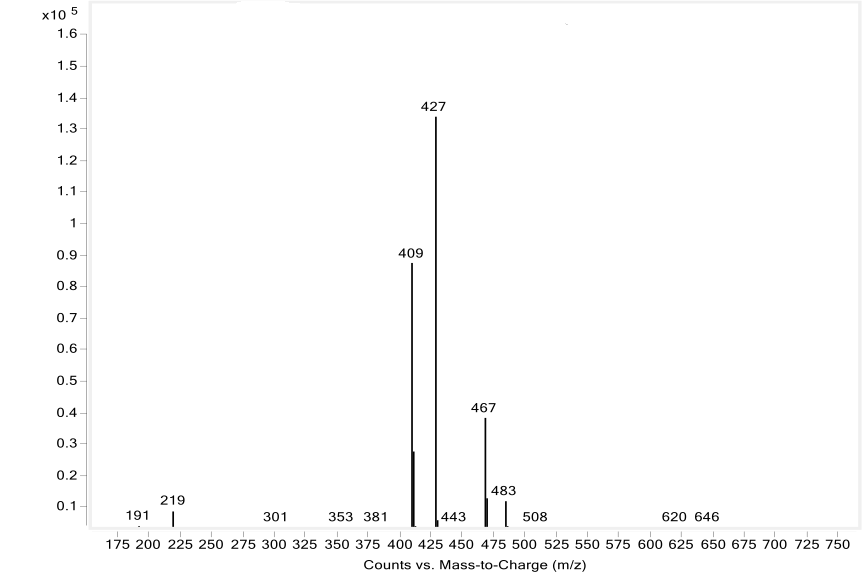


Supplementary Figure S4


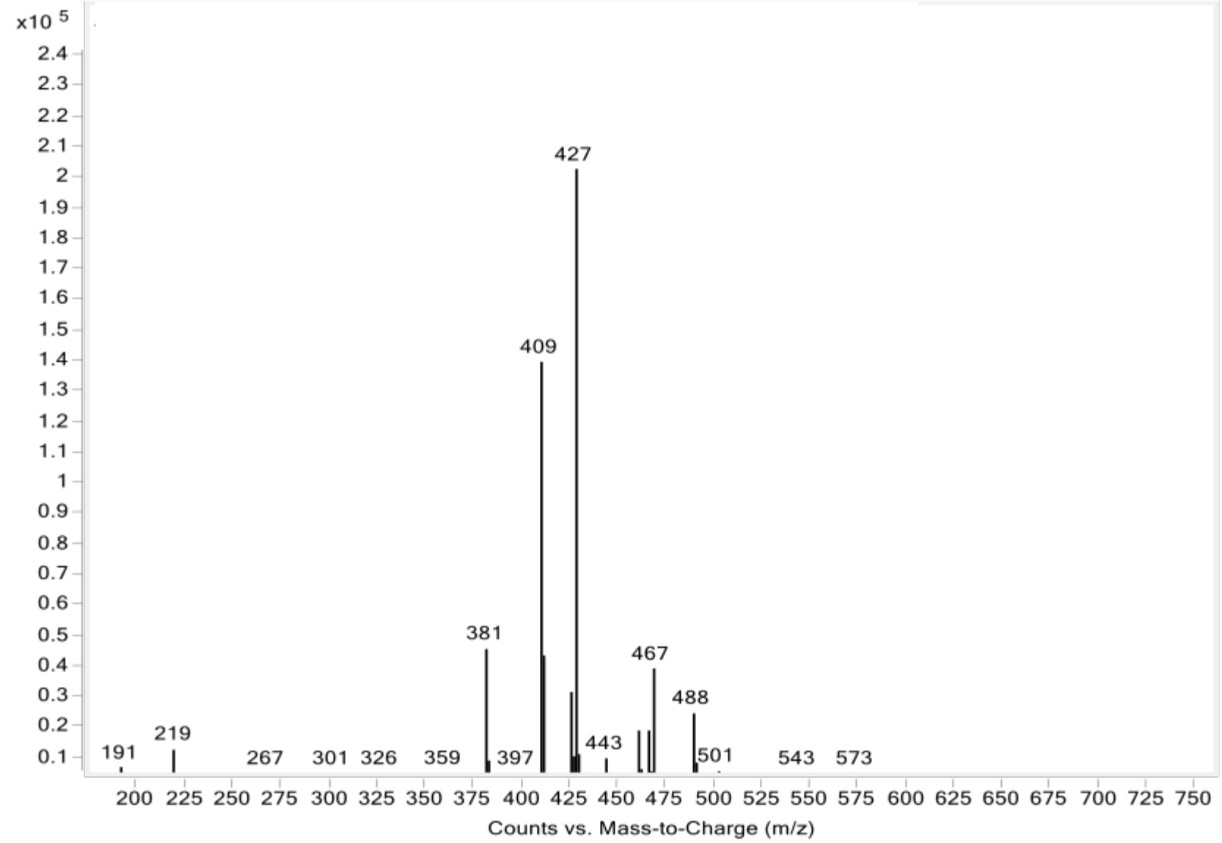


Supplementary Figure S5


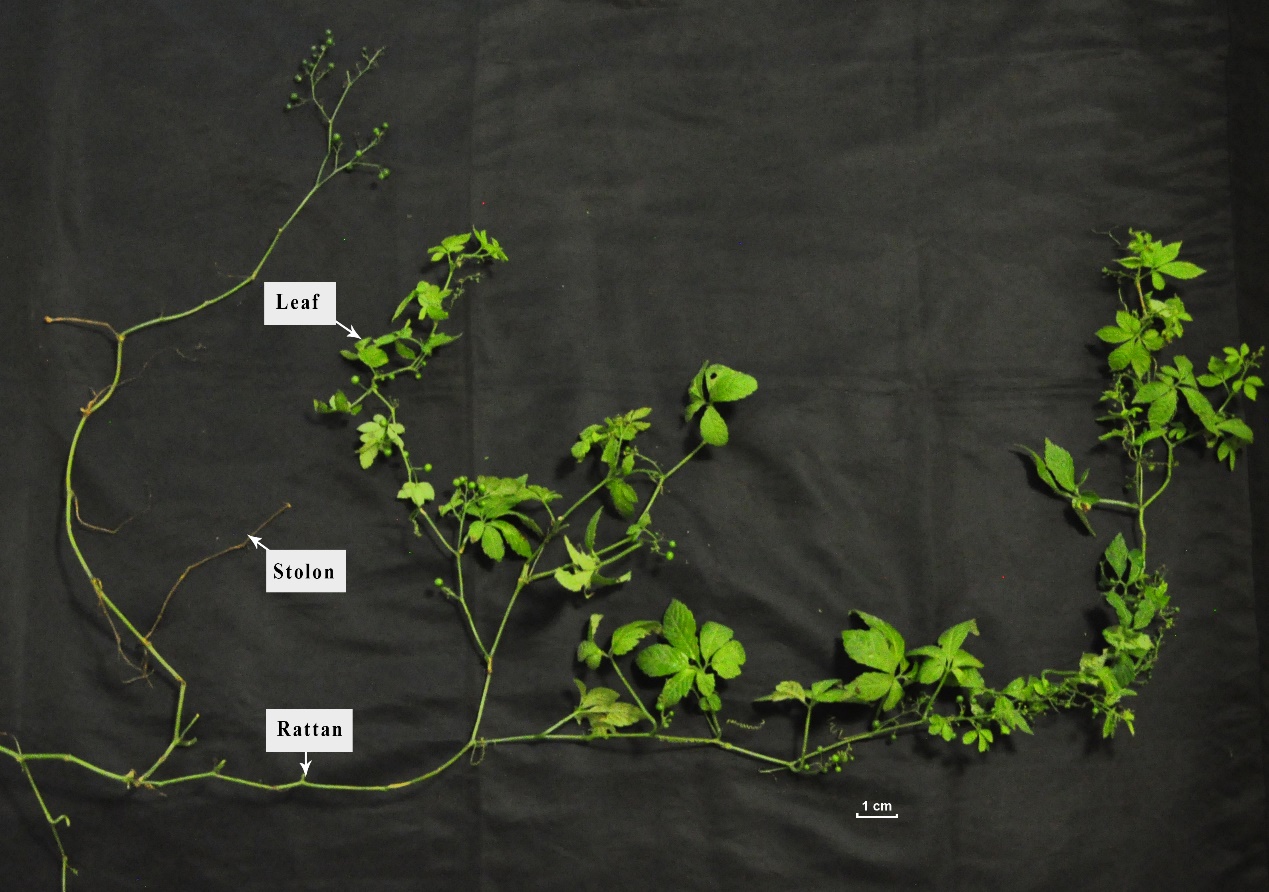

Supplement: Supplementary Figure S1 — (A) Functional classification by KEGG. The number of genes annotated to pathways, including Cellular Processes, Environmental Information Processing, Genetic Information Processing, and Metabolism. (B) Venn diagram of the KEGG, SwissProt, Pfam, and GO results for the G. longipes transcriptome. (C) Functional classification by GO. The number of genes annotated into the three categories: Biological Process, Cellular Component, and Molecular Function. [file Data_Sheet_1.docx]
